# Evaluation of a tailored implementation strategy for audit-generated improvements in perinatal care

**DOI:** 10.1136/bmjoq-2025-003421

**Published:** 2025-09-16

**Authors:** Ien van der Woerdt-Eltink, Andrea Drost, Gera Welker, Ageeth Rosman, Jan Jaap Erwich, Esteriek de Miranda

**Affiliations:** 1 Obstetrics and Gynecology, University Medical Centre Groningen, Groningen, Netherlands; 2 Institute for Healthcare, Rotterdam University of Applied Sciences, Rotterdam, Netherlands; 3 Centre of Expertise on Quality and Safety, University Medical Centre Groningen, Groningen, Netherlands

**Keywords:** perinatal audit, substandard care factors, tailored implementation strategy, behaviour change, quality improvement

## Abstract

**Background:**

Perinatal audit identifies substandard factors in perinatal care for quality improvement of care. However, not all identified improvement objectives achieve effective implementation. The ACTion method, using a 7-step Plan–Do–Check–Act cycle, was developed for local perinatal care professionals to enhance tailored implementation by interactive learning, training and coaching of implementation and behaviour change principles.

This study aimed to evaluate the efficacy of the ACTion method within all perinatal cooperation groups (PCGs) in the northern region of the Netherlands.

**Methods:**

A mixed-methods design was used for effect and process evaluation. Descriptive and inferential statistical methods were applied to analyse participants’ knowledge; skills; motivation; and the number, nature and implementation stage of improvement objectives. Additionally, influencing factors were examined through inductive thematic analysis.

**Results:**

A multidisciplinary ACTion team was formed in all 11 PCGs. From the initially 93 participating ACTion team members, 86% completed the full training.

Knowledge and skills after implementation of the ACTion method improved significantly, with mean scores increasing from 2.53 to 3.70 on a 1–5 Likert scale (p<0.001, r=0.9). ACTion teams addressed 3–19 improvement objectives, with implementation ranging from 14% to 67%, depending on time of start of the project and influencing factors. In total, 98 improvement objectives were addressed, of which 46 (47%) were fully implemented and secured. As emerged from interviews, a proactive key person and ongoing coaching during the follow-up phase were instrumental in driving these efforts. The multidisciplinary approach and collaborative efforts in regional obstetric care enhanced mutual understanding and cooperation across disciplines. Impeding factors included limited time, manpower and lack of commitment within the PCG.

**Conclusion:**

A locally tailored approach, involving interactive learning, training and coaching in the ACTion method, provides a valuable framework for implementing audit-driven improvement objectives in maternal and perinatal care, while simultaneously fostering interprofessional cooperation.

**WHAT IS ALREADY KNOWN ON THIS TOPIC?**

Perinatal audit identifies substandard factors in maternal and perinatal care in order to improve the quality of care. However, translating identified improvement objectives into effective actions often fails due to limited implementation strategies, highlighting the need for tailored approaches that support local professionals in implementing changes.

**WHAT THIS STUDY ADDS?**

The ACTion method—integrating interactive learning, training and coaching in implementation and behaviour change—strengthens implementation skills, fosters multidisciplinary collaboration and promotes structured implementation of a substantial part of improvement objectives, while also encouraging formalisation through integration into the perinatal audit organisation.

**HOW THIS STUDY MIGHT AFFECT RESEARCH, PRACTICE OR POLICY**

The findings highlight the need for training, tailored multidisciplinary strategies and proactive leadership for effective implementation. Policymakers and researchers can use these insights to address barriers like time and manpower constraints, enhancing the impact of perinatal audits on care quality. Though focused on perinatal care, these insights apply across the spectrum of medical disciplines.

## Introduction

 Perinatal audit aims to improve perinatal outcome by identifying substandard care factors in cases of severe adverse outcome, enhancing quality improvement.^1^,^5^ Audit is a cycle of identifying cases, data collection, analysing information, recommendation of solutions, implementation of changes, evaluation and redefining.^25^,^8^ According to the WHO guidance, recommendations should follow SMART criteria: specific, measurable, achievable, relevant and time-bound. Responsibility for monitoring progress should also be assigned.^5^ Effective implementation strategies are critical for completing the audit cycle.^7^,^9^

In comparison with other European countries, studies published in 2004 and 2008 have shown that the Netherlands had a relatively high perinatal mortality rate from a gestational age of 22 weeks onwards.^4 10 11^ Regional perinatal audits were introduced to address this issue, alongside interdisciplinary perinatal cooperation groups (PCGs) in order to foster collaboration.^4 12 13^ These PCGs organise local multidisciplinary perinatal audit meetings at least two times per year, reviewing anonymised cases of severe outcomes in a blame-free environment.^4 14^ By identifying substandard care factors, perinatal care professionals formulate improvement objectives to prevent recurrence. However, many objectives remain non-actioned, leading to recurrent discussions in subsequent audits.^2 14^

In the Netherlands, the state-funded foundation Perined processes the national registration of perinatal/childbirth data and oversees the coordination of perinatal audit (www.perined.nl). Local audit data, including improvement objectives, are recorded in the perinatal audit application (PAA) using standardised forms. Yet, very few objectives meet SMART criteria (specific, measurable, achievable, relevant and time-bound), underscoring challenges in formulating actionable objectives.^14^,^16^ Evidence indicates that locally developed objectives are more successfully implemented than top-down directives, emphasising the need for context-specific approaches.^4^

### ACTion method

**Figure 1:**
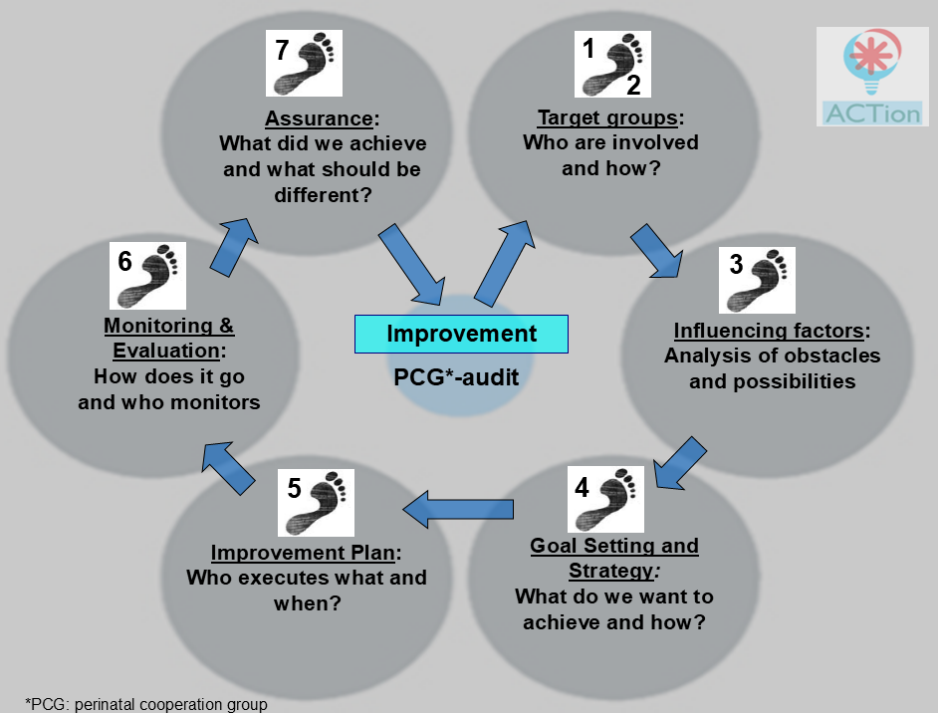
ACTion quality cycle.

To enhance the ability of local professionals to implement improvement objectives derived from perinatal audits, the ACTion project (Audit-generated Changes in Perinatal Care using Tailored Implementation Strategies) was initiated in 2013 in all PCGs of the northern region of the Netherlands. The project involved implementation of the ACTion method in each PCG, consisting of guided training in the use of the ‘ACTion Quality Cycle’ (figure 1; online supplemental material 1) by interactive learning,^17^ implementation knowledge and skills^18^ and behaviour change,^19^ supplemented with an implementation checklist (Netherlands),^20^ a Gantt chart^21^ and the Effective Practice and Organisation of Care (EPOC) taxonomy of implementation strategies.^22^ The ACTion Quality Cycle, a 7-step quality cycle based on Deming’s Plan–Do–Check–Act model,^23^ was developed with tailoring applied at two levels: (1) adaptation to local contextual factors to support implementation and (2) integration within the PCG collaboration structure through designated implementation teams and structured feedback loops to foster organisational learning. It served as a practical guideline for participants.

It was hypothesised that successful adoption of the ACTion method would improve the development and execution of local improvement objectives, contributing to improved quality of perinatal care. The aim of this mixed methods study was to evaluate the efficacy of the ACTion method.

## Methods

### Design

To evaluate the ACTion method, a mixed-methods action research design was employed^24^,^27^ (online supplemental material 2). The intervention was implemented in three phases according to a stepped-wedge cluster trial design^28^ (table 1), allowing insights from earlier groups to inform iterative refinement in subsequent ones. For example, the second group was advised to begin with a larger team composition to strengthen engagement.

**Table 1:** Timeline of the ACTion method: training and follow-up

	T1	T2	T3	T4
	Mar–Jul 2013	Sept–Dec 2013	Feb–Jun 2014	Jul 2014–Aug 2016
Group 1	Training	Follow-up	Follow-up	Follow-up
Group 2		Training	Follow-up	Follow-up
Group 3			Training	Follow-up

Data collection methods included paper-based questionnaires, semistructured interviews and structured observations. These instruments were selected to examine implementation processes and outcomes while allowing for triangulation and iterative adjustment of the intervention.^29 30^ Structured observations were conducted by all three ACTion trainers during each training session, with logs cross-checked to ensure investigator triangulation.^2631^,^34^ Semistructured interviews were carried out by either a trainer or an independent researcher to balance contextual insight with impartiality and minimise interviewer bias.^35^

To enhance the validity and reliability of findings, methodological triangulation was applied. Data triangulation was achieved by integrating observational field notes, validated questionnaires and semistructured interviews. These multiple data sources enabled cross-validation and provided a more comprehensive understanding of both the implementation process and contextual factors.

### Setting and study population

The ACTion project was conducted from March 2013 to August 2016 across all eleven PCGs participating in the regional knowledge network ‘Pregnancy and Childbirth North Netherlands’ in the northern region of the Netherlands (about 12 000 live births annually). Each PCG formed an ACTion team of local perinatal care professionals, including representatives from key professions such as obstetricians, community and/or hospital midwives, obstetric nurses, paediatricians and paediatric nurses, to implement improvement objectives identified during perinatal audits. The study population comprised members of the ACTion teams.

### Patient and public involvement

Patients and the public were not involved in the design, conduct, reporting or dissemination of this study.

### Intervention

The ACTion method, delivered by three trainers with complementary expertise (educational specialist, implementation expert and gynaecologist), consisted of three live interactive training sessions. During the first session, the ACTion quality cycle (figure 1) was explained, while in subsequent sessions, it served as a discussion framework. Each session started with behavioural change and implementation theory, followed by a flexible structure allowing teams to apply a local improvement objective from their perinatal audit. This adaptive approach aimed to bridge the gap between theory and practice.

### Training and follow-up

Following the ACTion quality cycle (figure 1), designing an appropriate and aligned implementation plan started with identifying the stakeholder groups relevant to each improvement objective and understanding their respective roles. The ACTion teams tailored the implementation plans by analysing local facilitating and impeding factors and choosing tailored implementation strategies to achieve the improvement objectives accordingly. Between training sessions, participants were assigned homework to further develop their implementation plans. After the three interactive training sessions, follow-up meetings were scheduled at least twice a year with ACTion teams. During these meetings, tailor-made coaching was provided to support implementation.

### Data collection

Data were collected through paper-based questionnaires completed by ACTion team members at baseline and follow-up, assessing demographic characteristics, motivation (based on the Multidimensional Work Motivation Scale^36 37^ and Self-Determination Theory),^38^ self-assessed implementation-related knowledge and skills, behaviour change and experiences with the ACTion method. Motivation was measured using a 5-point Likert scale (from ‘strongly disagree’ to ‘strongly agree’), while implementation-related knowledge and skills were self-assessed using a 5-point scale ranging from ‘low’ to ‘high’.

Semi-structured interviews were conducted with the ACTion team and PCG board members to explore perceived facilitators and barriers, dissemination of the ACTion method within the PCGs and local contextual factors. Additionally, board members completed a short questionnaire (multiple answers) on their involvement and collaboration within their PCG. Structured observations by trainers were used to identify facilitating and impeding factors, and to monitor the number and nature of improvement objectives addressed, as well as their application in obstetric care.

### Data analysis

**Figure 2:**
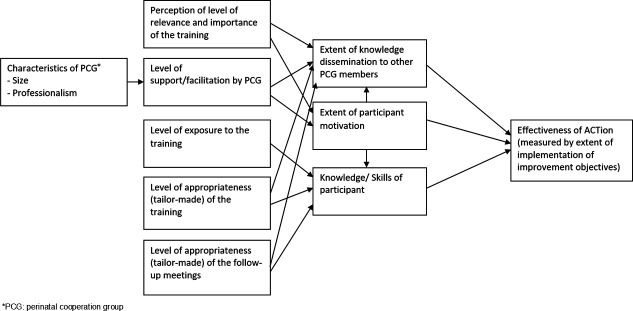
Conceptual model of the factors that may influence the effectiveness of ACTion method.

A conceptual model was developed to guide the evaluation of potential influencing factors and their relationship with the effectiveness of the ACTion project (figure 2). This model structured the analysis by defining key variables and guiding data interpretation, assessing both the project’s effectiveness and the factors influencing its implementation.

Descriptive statistics summarised baseline characteristics of PCGs; ACTion team members; and the number, nature and implementation stages of improvement objectives. Data are reported as frequencies (n, %), medians with IQRs or means (M) with SD where appropriate. Questionnaire data were checked for inconsistencies, outliers and missing values. Exploratory factor analysis grouped items related to motivation and to knowledge and skills, with Cronbach’s alpha values >0.70 indicating acceptable internal consistency. For normally distributed variables (assessed via histograms and the Kolmogorov–Smirnov test), paired t-tests were used to assess pre–post differences. To analyse individual change, difference scores (post–pre) were calculated for motivation and knowledge and skills. Multiple regression analysis was then used to explore associations between the difference scores on knowledge and skills and potential influencing factors such as intrinsic motivation, training appraisal and facilitation by PCG. As each participant contributed only one observation, assumptions of independence were met.

Qualitative interview data were analysed using an inductive thematic analysis approach.^39^ Two researchers independently transcribed and coded the data and then compared and discussed their findings. Code frequencies per participant and PCG were summarised in Microsoft Excel (Microsoft 2013). Relationships between influencing factors and implementation outcomes were explored and discussed by the trainers to identify key facilitators and barriers.

All quotes presented in the results section were translated from Dutch into English and independently verified by two bilingual researchers to preserve the original meaning.

### Ethical considerations

Based on the criteria of the Dutch Medical Research Involving Human Subjects Act (WMO), this study was outside this scope. The PCG board and its members were informed about the purpose of the ACTion study, after which individual healthcare providers were invited to join the ACTion teams. Written consent was obtained. Data were anonymised, with participant names stored separately from the data. Each participant received a unique identification code (ID), accessible only to researchers. This key was password-protected and stored in a secure digital environment of the University Medical Centre Groningen.

## Results

In all 11 PCGs in the northern region of the Netherlands, multidisciplinary ACTion teams were trained during one of three time periods (table 1). A total of 93 participants started the basic training, with 80 professionals across ten disciplines completing full training (86%) (table 2; Online supplemental material 3). Of the 93 participants who received the questionnaire at the start of the project, 72 (77%) completed the questionnaire. At the end of the project, 80 participants received the questionnaire, of which 56 (70%) returned the questionnaire. Pre- and post-test questionnaires from 55 participants (62%) were available for analysis.

**Table 2:** Participating professionals

Participants ACTion teams (n)	At start (n)	Stopped after first training (n)	Completed full training (n)
Community midwife	35	7	28
Hospital midwife	14		14
Obstetrician	12	2	10
Paediatrician	5	2	3
Obstetric nurse	9		9
Paediatric nurse	1		1
Nurse/midwife manager	11	1	10
Maternity care manager	4	1	3
General practitioner	1		1
Secretary*	1		1
Total	**93**	**13**	**80**

* Support ACTion team.

Observations and interviews revealed considerable variation between PCGs in terms of size, organisational characteristics, local structure, team composition and workforce shortages across participating PCGs. These differences significantly influenced how the ACTion method was adopted and operationalised in each setting, limiting the ability to identify consistent patterns or meaningful comparison of implementation outcomes across sites.

In group 1 (n=4), high dropout rates led to training being delivered in smaller teams, often lacking representation from all targeted professionals. This highlighted the vulnerability of small teams, particularly when members were unable to attend regularly. Commitment to implementation of improvements was also limited within some PCGs. In response, larger ACTion teams were formed in the second training period, to ensure broader representation. Dropout reasons included time pressure, lack of motivation, personal circumstances or insufficient understanding of the project’s objectives and the impact of their required commitment. ACTion team sizes ranged from 4 to 11 members (online supplemental material 3).

During the 3-year ACTion project, teams across all participating PCGs addressed 98 improvement objectives (range 3–19), with variation linked to the start date of the project and the number of priorities identified during perinatal audits (table 3). Of these, 46 objectives (47%; range 14%–64%) were successfully implemented and embedded in routine obstetric care, following the structured steps of the ACTion quality cycle (figure 1).

**Table 3:** Distribution of improvement objectives across steps of the ACTion quality cycle (figure 1; online supplemental material 1)

	Group 1 (n=4)				Group 2 (n=4)				Group 3 (n=3)			Total
	(Mar–Jul 2013)				(Sept–Dec 2013)				(Feb–Jun 2014)			
**ACTion team**	**A**	**B**	**C**	**D**	**E**	**F**	**G**	**H**	**I**	**J**	**K**	
Improvement objectives* (n)												
Steps 1 and 2	1	2	2		4	3			1	1		24
Step 3	8	2	1		1		1	2	1	1		17
Step 4	3					1		2				6
Step 5	1			4	1	3	3	1		1	1	15
Step 6	1	1	3	2			2		1		2	12
Step 7	5	1			1	12	5	8	1	1		34
**Total (n**)	**19**	**6**	**6**	**6**	**7**	**19**	**11**	**13**	**4**	**4**	**3**	**98**
Of which fully implemented and secured (steps 6 and 7) (n/%)	**6** (32%)	**2** (33%)	**3** (50%)	**2** (33%)	**1** (14%)	**12** (63%)	**7** (64%)	**8** (62%)	**2** (50%)	**1** (25%)	**2** (67%)	**46** (47%)

* The top 10 improvement objectives addressed by ACTion teams are listed in online supplemental material 4).

Observational data from follow-up meetings indicated that in several ACTion teams, PCG members joined ACTion to address newly selected improvement objectives. Their involvement was the result of intensified stakeholder engagement efforts, with professional backgrounds aligned to the specific focus of each improvement objective. This context-sensitive approach reflected principles of action research and supported the dissemination of knowledge and skills related to the application of the ACTion Quality Cycle.

ACTion teams led by a proactive key person, and actively involving PCG members outside the ACTion team, were most successful in implementing and embedding improvement objectives. All PCGs structurally integrated the ACTion method into the perinatal audit procedures and formalised it within their organisational agenda.

On a Likert scale of 1 to 5, participants reported an increase in their knowledge and skills regarding implementation following the basic training (M=2.53; SD=0.62 vs M=3.70; SD=0.56, p<0.001) (online supplemental material 5). Intrinsic motivation was the only significant predictor of baseline implementation-related knowledge and skills (β=0.388, p<0.01; 95% CI 0.066 to 0.555). Two predictors accounted for 27% of the variance in post-intervention knowledge and skill gains (R^2^=0.27, p<0.01): intrinsic motivation showed a significant negative association (β=−0.449, p<0.01, 95%CI −0.530 to −0.114), whereas appraisal of the training sessions showed a positive trend (β=0.281, p<0.056, 95%CI −0.009 to 0.690). Attendance at follow-up meetings was not a significant predictor (β=−0.039, p=0.787, 95%CI −0.451 to 0.344) (online supplemental material 6).

### Factors influencing the effectiveness of training and follow-up meetings

All PCGs actively participated in the ACTion project. Based on observations and interviews, several factors were identified that impacted the effectiveness of the ACTion method and follow-up meetings. Participants appreciated having the training and follow-up meetings at a local location. However, the 4–6-week interval between basic training sessions was often considered too long. Follow-up meetings were deemed necessary to supplement the basic training, especially for further development of knowledge and skills in implementation and change management, and to ensure progress in the implementation of improvement objectives.

’Fortunately, we had those follow-up meetings, and we continued working on the improvement points from the audit ourselves—but it was different from what I’d expected. I thought: OK, bring it on—how exactly does it work, and then we’ll just do it that way’. – Participant 13

The ACTion teams determined how to tailor the implementation process and strategy to the local context. The ACTion Quality Cycle (figure 1) served as a practical tool for applying and disseminating the strategy. According to interview findings, participants’ motivation was shaped by the perceived necessity to implement improvement objectives. This finding was validated by the questionnaire analysis, which demonstrated that the perceived relevance and applicability of these improvements positively affected participants’ intrinsic motivation to engage in the training (β=0.654, p<0.001, 95%CI 0.488 to 0.955) (online supplemental material 7). Monitoring of the seven steps in the ACTion quality cycle and celebrating successes, such as achieving (sub-)objectives within the ACTion team, positively impacted the progress of the implementation process. Conversely, nearly all participants cited major changes in obstetric care, such as maternity unit closures, as barriers to their motivation to engage in the ACTion project or implement improvements.

‘When I heard about it, I thought: “Oh great, here’s yet another thing that needs doing”. But we were all very clear—we really noticed that the issues identified in the audit were just being left unaddressed. So we all strongly agreed: “This just isn’t acceptable—something really does need to be done about it”. But then nothing actually happened, or only for a short while. And then it would fizzle out again. So yes, we all recognised the value of it—myself included’. – Participant 15

Interview data indicated that at the collaboration level, a proactive key person—who took the lead—was essential for sustaining progress and continuity in implementing improvement objectives. This individual facilitated engagement by motivating colleagues, demonstrating perseverance and driving progress. Additionally, interviewees emphasised the critical role of the ACTion project coordinator—responsible for training and coaching teams—in advancing implementation processes. Participants further highlighted that the multidisciplinary approach and regional collaboration in obstetric care fostered mutual understanding, strengthened cooperation and increased support for the proposed improvement plans. Key barriers to effective cooperation within the ACTion team included time constraints, workforce shortages and limited support from PCG leadership and/or members, which also affected the progress, evaluation and embedding of improvement objectives within the PCG.

‘We work with an annual schedule, and that works really well. We have secretarial support—the secretary supports both our ACTion and perinatal audit meetings, takes the minutes, organises all the meetings and arranges the lunches. That’s really helpful. It means you know exactly when the meetings will take place throughout the year’. – Participant 14

‘The more support you have for wanting to take something on, the easier it gets—because then the paediatrician says: oh, we could do this, and the social worker says: we could do that. It really shows that when you collaborate across different disciplines, it’s much easier than when you try to make changes within just one professional group—unless it’s purely a topic that only concerns obstetrics. What we’re increasingly finding is that topics often have different aspects, where we overlap with other disciplines’. – Participant 7

Overall, findings align with the conceptual model, confirming that participant motivation, training exposure and organisational support play a critical role in implementation success.

## Discussion

This study evaluated the effectiveness of the ACTion method, which integrates training and coaching to enhance the implementation of perinatal care recommendations. The method demonstrated an increase in implementation knowledge and skills, with 47% of improvement objectives implemented during the research period. Consistent with the conceptual model, intrinsic motivation, training exposure and organisational support emerged as key determinants of implementation outcomes. Intrinsic motivation positively predicted baseline knowledge and skills, but was negatively associated with post-training gains. Leadership by key team members, supported by trainers, played an instrumental role in driving progress. The multidisciplinary approach facilitated collaboration among perinatal healthcare professionals, strengthening knowledge dissemination and embedding improvement objectives into routine practice. However, structural barriers, including time constraints and contextual characteristics, negatively affected engagement. Limited commitment from PCG stakeholders hindered further progress, reinforcing the model’s emphasis on organisational support. While follow-up meetings were considered essential, their direct impact on skill acquisition was limited. Systematic monitoring and celebrating successes positively influenced implementation efforts. The ACTion method was successfully embedded into perinatal audits and integrated into the PCG agendas of the participating PCGs, reinforcing its potential for sustainable implementation in perinatal care.

In contrast to other high-income countries, the Netherlands adopted a bottom-up approach to implement recommendations from perinatal audits in obstetric care. This study represents the first evaluation of the ACTion method in a high-income country, designed to train and support local obstetric healthcare providers in implementing improvement objectives.^2^ As all 11 PCGs in the northern region of the Netherlands participated in the ACTion project, the entire region was represented, ensuring that the results are highly generalisable to this region.

Another important feature of this study is its grounding in participatory action research (PAR), which allowed for a bottom-up, iterative approach tailored to local contexts.^27 40^ This contributed to stakeholder engagement, contextual relevance and opportunities for interprofessional learning. Literature suggests that PAR can support system learning and adaptive capacity when applied thoughtfully.^41 42^ The stepwise implementation enabled feedback-informed adjustments between teams, which aligns with Lockwood *et al*^43^, who emphasise the importance of involving frontline professionals in shaping improvement strategies.^43^ The ACTion project supported professionals in developing job crafting and self-management competencies,^44^ and the integration of training within real practice contexts contributed to enhanced professional autonomy and team-based learning.^45^ Additionally, such approaches require sufficient time, trust and shared purpose to be successful.^46^ The interprofessional components of the intervention are in line with evidence suggesting that collaborative learning improves both team functioning and quality of care.^47 48^

The proportion of implemented improvement objectives prior to the ACTion project is unknown, as this was not systematically recorded yet. We found 47% of improvement objectives implemented. This proportion appears higher than in a hospital-based audit study reporting 21% implementation within 15 months, where department heads—together with clinical staff—were responsible for executing improvement plans.^7^ In our 3-year project, implementation was approached more bottom-up, and full implementation appeared more likely when a proactive key person led the process and PCG professionals were actively involved—particularly when ACTion team members engaged colleagues in applying the ACTion method and associated ACTion quality cycle.

Consistent with self-determination theory (SDT), which emphasises the role of autonomous motivation in learning and behavioural change, intrinsic motivation significantly predicted baseline implementation-related knowledge and skills.^49^ However, it was negatively associated with post-intervention gains, possibly reflecting a ceiling effect among already motivated participants. This aligns with previous SDT research suggesting that individuals with high autonomous motivation often show proactive engagement at baseline, leaving less room for further improvement.^50 51^ Participants nonetheless experienced a significant increase in implementation knowledge and skills following the training, which influenced their ability to integrate improvements into clinical practice. This finding supports research demonstrating the effectiveness of local, context-specific interventions in enhancing healthcare delivery.^2 52^

The inverse association between intrinsic motivation and observed gains may partly be explained by the use of self-assessed outcomes. While practical, such self-assessments may introduce response-shift bias, as participants’ internal standards may evolve during the intervention.^51 53^ To strengthen the validity of findings, this study applied methodological and data triangulation, in line with best practices in mixed-methods and implementation research. Triangulation—by integrating observational field notes, validated survey data and semistructured interviews—helped reduce single-method bias and enabled cross-validation and contextual interpretation of the findings.^54^

Interviewees indicated that follow-up meetings after basic training were necessary for further developing implementation knowledge, skills and change management. However, this effect was not confirmed by the quantitative analysis, with a quarter of the variance in participants’ learning outcomes explained by two factors: intrinsic motivation and appraisal of the training sessions. The remaining variance, attributed to unidentified factors, suggests the need for further research to identify additional influences on learning outcomes. This finding is consistent with evidence showing that effective learning requires both targeted interventions and favourable contextual conditions.^6 55 56^

### Limitations of the study

The organisation of obstetric care in the Netherlands, where midwives provide the majority of obstetric care in a primary-care setting, limits the applicability of our findings to other healthcare systems. As Eskes *et al*^4^ noted in their evaluation of perinatal care audits across Europe,^4^ variation in healthcare structures poses significant challenges for generalisability. Similarly, Winters^57^ emphasised the critical role of regional context, organisational culture, leadership and structure in driving client-focused quality improvement.^57^ It is plausible that our experiences with local teams could be transferable to other settings where professionals work collaboratively together.^58^

Nonetheless, an important consideration in interpreting the findings is the substantial variation between participating PCGs. Contextual differences influenced how the ACTion method was adopted and implemented, thereby limiting the extent to which its effectiveness could be systematically assessed in relation to specific contextual characteristics. While this heterogeneity enhances ecological validity and reflects the complexity of real-world practice, it also constrained the ability to draw consistent conclusions about the contextual drivers of implementation success.

All PCGs in the northern region of the Netherlands that participated in this ACTion project had previously been involved in a study aiming to implement unit-based perinatal mortality audit.^15^ Since PCG board and ACTion team members were informed of the objectives, this prior involvement may have introduced a Hawthorne effect.^59^ An independent researcher participated in the interviews, but ACTion trainers were primarily responsible for conducting the research. This may have led to interviewer or courtesy bias, making participants hesitant to share honest opinions. Self-reported gains in knowledge and skills may also reflect response bias, potentially amplified by familiarity between trainers and participants—possibly leading to an overestimation of the ACTion method’s effectiveness.^50 60^

Although measures were implemented to minimise researcher bias—such as involving an independent researcher, engaging multiple trainers and critically discussing results—potential influence of the Rosenthal effect cannot be excluded.^53 61^ Finally, PCG members volunteered for the ACTion project, and no randomised selection of team members was applied. Most volunteers were highly motivated to address recurring improvement objectives identified in perinatal audits. Consequently, volunteer bias should be considered when interpreting the findings, as it may have led to an overestimation of the results.

While the ACTion project included structured follow-up meetings and coaching over 3 years, this does not guarantee sustainability. Sustaining improvements requires more than temporary support; it demands integration into daily workflows, organisational culture and leadership structures.^7 62^ Similarly, continued adaptation, stakeholder commitment and monitoring are essential for maintaining complex interventions.^63^

Despite promising short-term effects, the extent to which the ACTion method and its related improvements are maintained beyond the project period remains unclear. As noted in our recommendations, a follow-up study is warranted to assess the long-term impact of the intervention on care quality and outcomes. In addition, further research could explore the organisational and contextual conditions under which sustainability is most likely to occur.

## Conclusion

A locally tailored approach of interactive learning combined with training and coaching of multidisciplinary audit teams in the ACTion method offers a valuable framework for the implementation of audit generated improvement objectives in perinatal care with stimulation of interprofessional cooperation being experienced as positive side effect.

### Recommendations

Healthcare systems should invest in continued training and coaching for multidisciplinary teams to equip them with the knowledge and skills to implement change in perinatal care.

A PCG-supported proactive key person who is willing to take the lead and an engaged trainer are essential to drive progress, sustain motivation and integrate changes into routine practice. Addressing structural barriers, such as time constraints, workforce shortages and organisational commitment, is critical for effective cooperation within the multidisciplinary teams. Strengthening collaboration and securing leadership buy-in will improve the feasibility and impact of quality improvement efforts.

Embedding the ACTion method into perinatal audits and PCG agendas may ensure its long-term sustainability. Aligning this approach with institutional priorities supports lasting quality improvements. Further research is needed to assess its long-term sustainability and scalability to other regions or healthcare settings. Additionally, a follow-up study is recommended to evaluate the sustained impact of the implemented changes on maternal and perinatal outcomes.

## Supplementary material

10.1136/bmjoq-2025-003421 online supplemental file 1

10.1136/bmjoq-2025-003421 online supplemental file 2

10.1136/bmjoq-2025-003421 online supplemental file 3

10.1136/bmjoq-2025-003421 online supplemental file 4

10.1136/bmjoq-2025-003421 online supplemental file 5

10.1136/bmjoq-2025-003421 online supplemental file 6

10.1136/bmjoq-2025-003421 online supplemental file 7

## Data Availability

Data are available upon reasonable request. All data relevant to the study are included in the article or uploaded as supplementary information.
