# Supplementary material for: Evaluation of a tailored implementation strategy for audit-generated improvements in perinatal care
Source: BMJ Open Qual. 2025 Sep 16;14(3):e003421. doi: 10.1136/bmjoq-2025-003421 (PMC12443171; doi:10.1136/bmjoq-2025-003421)
Supplement: online supplemental file 1 [file bmjoq-14-3-s001.docx]

**Supplemental material 1. Steps of the ACTion Quality Cycle**

| [**Step**](#stap1) **1 Preparing the change**   1. Assemble the stakeholders group. 2. Map out the original question or problem with stakeholders. 3. Check with the stakeholders whether the proposed change can actually solve the problem. 4. Map out the possible advantages and disadvantages of the change for each group of users, and use this information when choosing and making your change approach.   **Tools: 80/20 rule** [64], **fishbone** **diagram** [65], **brainstorming**, **8-step plan from Kotter** [66]. |
| --- |
| **Step 2 Define the objective**   1. Ensure that the objective to be achieved with the change is formulated using the SMART criteria, which stands for specific, measurable, acceptable, realistic, and time-bound.   **Tools: How to formulate SMART objectives**, **balanced score card**. |
| **Step 3 Study influencing factors**   1. Check which target groups there are and determine the needs per target group. 2. Find out who is the leader within the target group, who are the forerunners and those who lag behind and where the middle group is. 3. Analyse, where possible together with the target group, the setting of the implementation. Find out which factors hinder implementation (impeding factors or barriers) or help accelerate it (enhancing factors).   **Tools: Target group analysis, focus group, stakeholder analysis, overview of influencing factors, strengths/weaknesses analysis, implementation checklist** **National Health Care Institute Netherlands** [20]**.** |
| **Step 4 Determine an implementation strategy**  Choose implementation strategies that make use of facilitators and try to remove any impediments that may hinder progress.  **Tools: Intervention mapping**, **categories of implementation strategies, EPOC taxonomy of strategies** [22]**.** |
| **Step 5 Develop an implementation plan**   1. Write down the implementation plan 2. Discuss the plan with the stakeholders and ensure agreement. 3. Ensure that everyone involved in the implementation knows what is expected of them and has access to all necessary resources. 4. Discuss who to contact, when, and how in case of possible problems.   **Tools: Format implementation plan**, **time phasing: Gantt chart** [21], **PDCA cycle.** |
| **Step 6 Monitor and evaluate implementation**   1. Clearly define how, when, and by whom the progress of the implementation will be measured. 2. Continuously evaluate the implementation to make necessary adjustments to the plan in a timely manner. Additionally, collect feedback from those involved and provide feedback on the results of the evaluation.   **Tools: PDCA cycle**, **monitoring**, **process** **evaluation**. |
| **Step 7 Securing the change**  Continue to pay attention to the implementation. Ensure that the new working-method is included in the quality policy/guideline/protocol and continue to monitor and evaluate.  **Tools: Secure checklist in the organization**. |
