# Supplementary material for: Evaluation of a tailored implementation strategy for audit-generated improvements in perinatal care
Source: BMJ Open Qual. 2025 Sep 16;14(3):e003421. doi: 10.1136/bmjoq-2025-003421 (PMC12443171; doi:10.1136/bmjoq-2025-003421)
Supplement: online supplemental file 2 [file bmjoq-14-3-s002.docx]

**Supplemental file 2. Evaluation schedule ACTion method**

To evaluate the ACTion method, a mixed-methods action research design, a range of instruments were selected to study both the processes and outcomes of the intervention.

| **Measures** | **T1 (Group 1)**  **(March-Aug 2013)** | **T2 (Group 2)**  **(Sept-Dec 2013)** | **T3 (Group 3)**  **(Feb-June 2014)** | **T4**  **(July 2014-April 2016)** |
| --- | --- | --- | --- | --- |
| Characteristics of ACTion team members | Questionnaires | | | Interviews: two team members of each ACTion team |
| Characteristics of the PCGs | Log by project leader | | |  |
| Motivation individual participants | Pre-training questionnaire | | | Questionnaire 18 months after start intervention  Interviews with participants |
| Knowledge and skills | Questionnaire: self-assessment by individual participants | | |  |
| Experience with the intervention | Questionnaire completed by individual participants after training | | | Interviews: two team members of each ACTion team |
| Effectiveness of the intervention | Through monitoring and observation during training and follow-up meetings, interviews with ACTion team members and PCG board members, data collected by researchers. | | | |
