# Supplementary material for: Evaluation of a tailored implementation strategy for audit-generated improvements in perinatal care
Source: BMJ Open Qual. 2025 Sep 16;14(3):e003421. doi: 10.1136/bmjoq-2025-003421 (PMC12443171; doi:10.1136/bmjoq-2025-003421)
Supplement: online supplemental file 3 [file bmjoq-14-3-s003.docx]

**Supplemental file 3. Participating professionals per ACTion team**

|  | **Group 1 (n=4)** | | | | **Group 2 (n=4)** | | | | **Group 3 (n=3)** | | | **Total** |
| --- | --- | --- | --- | --- | --- | --- | --- | --- | --- | --- | --- | --- |
|  | (Mar-July 2013) | | | | (Sept-Dec 2013) | | | | (Feb-June 2014) | | |  |
| **ACTion team/PCG** | **A** | **B** | **C** | **D** | **E** | **F** | **G** | **H** | **I** | **J** | **K** |  |
| Participants profession (n) |  |  |  |  |  |  |  |  |  |  |  |  |
| Primary care midwife | 4 (1*^§^*) | 4 (1*^§^*) | 2 | 3 | 2 (1*^§^*) | 2 | 3 (1*^§^*) | 2 | 2 | 8 (2*^§^*) | 3 (1*^§^*) | 35 (7*^§^*) |
| Hospital midwife | 2 | 1 |  | 1 | 2 | 1 | 2 | 1 | 2 | 1 | 1 | 14 |
| Obstetrician | 1 (1*^§^*) | 1 | 1 | 1 (1*^§^*) | 1 | 1 | 1 | 2 | 1 | 1 | 1 | 12 (2*^§^*) |
| Paediatrician |  |  | 1 (1*^§^*) |  | 1 | 1 | 1 (1*^§^*) |  | 1 |  |  | 5 (2*^§^*) |
| Obstetric nurse | 1 | 1 |  | 1 |  | 2 | 1 |  | 1 | 1 | 1 | 9 |
| Paediatric nurse |  | 1 |  |  |  |  |  |  |  |  |  | 1 |
| Nurse/midwife manager | 1 (1*^§^*) |  | 1^*^ | 1 | 2 | 1 |  | 1 | 2 | 1 | 1^*^ | 11 (1*^§^*) |
| Maternity care manager |  |  | 1 (1*^§^*) |  | 1 |  |  |  |  | 1 | 1 | 4 (1*^§^*) |
| General practitioner |  |  |  |  | 1 |  |  |  |  |  |  | 1 |
| Secretary |  |  |  |  | 1 |  |  |  |  |  |  | 1 |
| **Total participants at start (n)** | 9 | 8 | 6 | 7 | 11 | 8 | 8 | 6 | 9 | 13 | 8 | 93 |
| ^§^ Stopped after 1st training | 3 | 1 | 2 | 1 | 1 |  | 2 |  |  | 2 | 1 | 13 |
| **Total participants completed full training (n)** | **6** | **7** | **4** | **6** | **10** | **8** | **6** | **6** | **9** | **11** | **7** | **80** |
