# Supplementary material for: Evaluation of a tailored implementation strategy for audit-generated improvements in perinatal care
Source: BMJ Open Qual. 2025 Sep 16;14(3):e003421. doi: 10.1136/bmjoq-2025-003421 (PMC12443171; doi:10.1136/bmjoq-2025-003421)
Supplement: online supplemental file 4 [file bmjoq-14-3-s004.docx]

**Supplemental file 4. Top 10 improvement objectives addressed by ACTion teams**

The most frequently addressed improvement objectives included:

1. Accurate CTG interpretation and the use of fetal monitoring protocols (8 teams)
2. Interprofessional communication across care settings (6 teams)
3. Differentiating between maternal and fetal heart rate (5 teams)
4. Timely paediatric involvement and structured clinical handover (5 teams)
5. Storage of the placenta for pathological examination (4 teams)
6. Documentation of time intervals and timeliness of perinatal actions (4 teams)
7. Instructions to patients on when to contact maternity/perinatal care providers (3 teams)
8. Detection and management of fetal growth restriction (3 teams)
9. Consistency and accuracy of documentation practices (3 teams)
10. Clinical response to decreased fetal movements (3 teams)
