# Supplementary material for: Evaluation of a tailored implementation strategy for audit-generated improvements in perinatal care
Source: BMJ Open Qual. 2025 Sep 16;14(3):e003421. doi: 10.1136/bmjoq-2025-003421 (PMC12443171; doi:10.1136/bmjoq-2025-003421)
Supplement: online supplemental file 5 [file bmjoq-14-3-s005.docx]

**Supplemental file 5. Knowledge and Skills - Pre-Post-test_SPSS**

Results analyses ACTion project Knowledge and Skills Pre-Post-test.

Paired samples T-test

The participants reported significantly higher levels of Skills and /Knowledge on the post-test ( = 3.70, SD = .56) than on the Pre-test (M = 2.53, SD = .62), t(54) = -15.537, p < .001, r = .90. This considered to be a very large affect size (67).

| **Paired Samples Statistics** | | | | | |
| --- | --- | --- | --- | --- | --- |
|  | | Mean | N | Std. Deviation | Std. Error Mean |
| Pair 1 | PPVgem | 2,5339 | 55 | ,61862 | ,08342 |
|  | PPNgem | 3,7021 | 55 | ,55706 | ,07511 |

| **Paired Samples Correlations** | | | | |
| --- | --- | --- | --- | --- |
|  | | N | Correlation | Sig. |
| Pair 1 | PPVgem & PPNgem | 55 | ,554 | ,000 |

| **Paired Samples Test** | | | | | | | | | |
| --- | --- | --- | --- | --- | --- | --- | --- | --- | --- |
|  | | Paired Differences | | | | | t | df | Sig. (2-tailed) |
|  |  | Mean | Std. Deviation | Std. Error Mean | 95% Confidence Interval of the Difference | |  |  |  |
|  |  |  |  |  | Lower | Upper |  |  |  |
| Pair 1 | PPVgem - PPNgem | -1,16818 | ,55759 | ,07519 | -1,31892 | -1,01744 | -15,537 | 54 | ,000 |

Effect size PP: √(-15,537^2^/(-15,537^2^+54)= √ 241,398369/ 295,398369=**.90**

Cohen, J. Statistical Power Analysis for the behavioral Sciences. 2nd ed. New York: Lawrence Erlbaum Associates; 1988. 579 p. Available: https://www.utstat.toronto.edu/brunner/oldclass/378f16/readings/CohenPower.pdf
