# Supplementary material for: Evaluation of a tailored implementation strategy for audit-generated improvements in perinatal care
Source: BMJ Open Qual. 2025 Sep 16;14(3):e003421. doi: 10.1136/bmjoq-2025-003421 (PMC12443171; doi:10.1136/bmjoq-2025-003421)
Supplement: online supplemental file 6 [file bmjoq-14-3-s006.docx]

**Supplemental file 6. Knowledge and Skills in relation to intrinsic motivation and appraisal of the training sessions**

Effect of the training: difference in knowledge and skills (pre- and post-measurement - PPVPPNdif)

The results of the multiple regression analysis indicated that two predictors explained 27% of variance of the participants’ Gain in Knowledge and Skills (PPVPPNdif) (R2=.274, F(3,36)=4.538, p<.01). Intrinsic Motivation (MOTint) was found to significantly predict PPVPPNdif (β= -.449, p<.01). Appraisal of the Training Sessions (EEgem) was found to be approaching significance as an predictor (β=.228, p<.10). Attendance was not found to significantly predict MOTint (β= -.039, n.s.).

| **Model Summary** | | | | | | | | | | | | | | | |  |
| --- | --- | --- | --- | --- | --- | --- | --- | --- | --- | --- | --- | --- | --- | --- | --- | --- |
| Model | R | | R Square | | Adjusted R Square | | Std. Error of the Estimate | | Change Statistics | | | | | | | |
|  |  |  |  |  |  |  |  |  | R Square Change | | F Change | | df1 | df2 | Sig. F Change | |
| 1 | ,524a | | ,274 | | ,214 | | ,49139 | | ,274 | | 4,538 | | 3 | 36 | ,008 | |
| a. Predictors: (Constant), Attendance, EEgem, MOTint  b. Dependent Variable: PPVPPNdif | | | | | | | | | | | | | | | | |
| **ANOVA^a^** | | | | | | | | | | | | | | | |  |
| Model | | | | Sum of Squares | | df | | Mean Square | | F | | Sig. | | | |  |
| 1 | | Regression | | 3,287 | | 3 | | 1,096 | | 4,538 | | ,008b | | | |  |
|  |  | Residual | | 8,693 | | 36 | | ,241 | |  | |  | | | |  |
|  |  | Total | | 11,980 | | 39 | |  | |  | |  | | | |  |
| a. Dependent Variable: PPVPPNdif | | | | | | | | | | | | | | | |  |
| b. Predictors: (Constant), Attendance, EEgem, MOTint | | | | | | | | | | | | | | | |  |

| **Coefficients^a^** | | | | | | | | | | | | | |
| --- | --- | --- | --- | --- | --- | --- | --- | --- | --- | --- | --- | --- | --- |
| Model | | Unstandardized Coefficients | | Standardized Coefficients | t | Sig. | 95,0% Confidence Interval for B | | Correlations | | | Collinearity Statistics | |
|  |  | B | Std. Error | Beta |  |  | Lower Bound | Upper Bound | Zero-order | Partial | Part | Tolerance | VIF |
| 1 | (Constant) | 1,636 | ,777 |  | 2,106 | ,042 | ,061 | 3,211 |  |  |  |  |  |
|  | MOTint | -,322 | ,103 | -,449 | -3,139 | ,003 | -,530 | -,114 | -,442 | -,464 | -,446 | ,985 | 1,016 |
|  | EEgem | ,340 | ,172 | ,281 | 1,973 | ,056 | -,009 | ,690 | ,260 | ,312 | ,280 | ,997 | 1,003 |
|  | Attendance | -,053 | ,196 | -,039 | -,272 | ,787 | -,451 | ,344 | -,081 | -,045 | -,039 | ,985 | 1,015 |
| a. Dependent Variable: PPVPPNdif | | | | | | | | | | | | | |

**Knowledge and Skills at start of ACTion project (PPV):**

| **Model Summary^b^** | | | | | | | | | | |
| --- | --- | --- | --- | --- | --- | --- | --- | --- | --- | --- |
| Model | R | R Square | Adjusted R Square | Std. Error of the Estimate | Change Statistics | | | | | Durbin-Watson |
|  |  |  |  |  | R Square Change | F Change | df1 | df2 | Sig. F Change |  |
| 1 | ,414^a^ | ,171 | ,104 | ,58103 | ,171 | 2,548 | 3 | 37 | ,071 | 1,680 |
| a. Predictors: (Constant), MOTint, EEgem, Attendance | | | | | | | | | | |
| b. Dependent Variable: PPVgem | | | | | | | | | | |

| **ANOVA^a^** | | | | | | |
| --- | --- | --- | --- | --- | --- | --- |
| Model | | Sum of Squares | df | Mean Square | F | Sig. |
| 1 | Regression | 2,581 | 3 | ,860 | 2,548 | ,071^b^ |
|  | Residual | 12,491 | 37 | ,338 |  |  |
|  | Total | 15,072 | 40 |  |  |  |
| a. Dependent Variable: PPVgem | | | | | | |
| b. Predictors: (Constant), MOTint, EEgem, Attendance | | | | | | |

| Coefficients^a^ | | | | | | | | | | | | | |
| --- | --- | --- | --- | --- | --- | --- | --- | --- | --- | --- | --- | --- | --- |
| Model | | Unstandardized Coefficients | | Standardized Coefficients | t | Sig. | 95,0% Confidence Interval for B | | Correlations | | | Collinearity Statistics | |
|  |  | B | Std. Error | Beta |  |  | Lower Bound | Upper Bound | Zero-order | Partial | Part | Tolerance | VIF |
| 1 | (Constant) | 1,006 | ,916 |  | 1,098 | ,279 | -,850 | 2,861 |  |  |  |  |  |
|  | Attendance | -,046 | ,232 | -,030 | -,199 | ,843 | -,516 | ,423 | ,023 | -,033 | -,030 | ,984 | 1,016 |
|  | EEgem | ,183 | ,204 | ,135 | ,898 | ,375 | -,230 | ,596 | ,151 | ,146 | ,134 | ,997 | 1,003 |
|  | MOTint | ,310 | ,121 | ,388 | 2,571 | ,014 | ,066 | ,555 | ,391 | ,389 | ,385 | ,983 | 1,017 |
| a. Dependent Variable: PPVgem | | | | | | | | | | | | | |

***Intrinsic Motivation is the only significant and strongest predictor for pre-measurement***
