# Supplementary material for: Evaluation of a tailored implementation strategy for audit-generated improvements in perinatal care
Source: BMJ Open Qual. 2025 Sep 16;14(3):e003421. doi: 10.1136/bmjoq-2025-003421 (PMC12443171; doi:10.1136/bmjoq-2025-003421)
Supplement: online supplemental file 7 [file bmjoq-14-3-s007.docx]

**Supplemental file 7. Intrinsic motivation**

**Motivation**

The results of the multiple regression analysis indicated that two predictors explained 60% of variance of the participants’ Instrinsic Motivation (MOTint) (R2 =.598, F(2,59)=43.845, p<.001). Relevance and applicability (MOTrn) was found to significantly predict MOTint (β=.654, p<.001). Facilitation (MOTfac) was not found to significantly predict MOTint (β=.173, n.s.).

| **Model Summary** | | | | | | | | | | | | | | | |  |
| --- | --- | --- | --- | --- | --- | --- | --- | --- | --- | --- | --- | --- | --- | --- | --- | --- |
| Model | R | | R Square | | Adjusted R Square | | Std. Error of the Estimate | | Change Statistics | | | | | | | |
|  |  |  |  |  |  |  |  |  | R Square Change | | F Change | | df1 | df2 | Sig. F Change | |
| 1 | ,773^a^ | | ,598 | | ,584 | | ,44564 | | ,598 | | 43,845 | | 2 | 59 | ,000 | |
| a. Predictors: (Constant), MOTfac, MOTrn | | | | | | | | | | | | | | | | |
| **ANOVA^a^** | | | | | | | | | | | | | | | |  |
| Model | | | | Sum of Squares | | df | | Mean Square | | F | | Sig. | | | |  |
| 1 | | Regression | | 17,415 | | 2 | | 8,707 | | 43,845 | | ,000^b^ | | | |  |
|  |  | Residual | | 11,717 | | 59 | | ,199 | |  | |  | | | |  |
|  |  | Total | | 29,131 | | 61 | |  | |  | |  | | | |  |
| a. Dependent Variable: MOTint | | | | | | | | | | | | | | | |  |
| b. Predictors: (Constant), MOTfac, MOTrn | | | | | | | | | | | | | | | |  |

| **Coefficients^a^** | | | | | | | | | | | | | |
| --- | --- | --- | --- | --- | --- | --- | --- | --- | --- | --- | --- | --- | --- |
| Model | | Unstandardized Coefficients | | Standardized Coefficients | t | Sig. | 95,0% Confidence Interval for B | | Correlations | | | Collinearity Statistics | |
|  |  | B | Std. Error | Beta |  |  | Lower Bound | Upper Bound | Zero-order | Partial | Part | Tolerance | VIF |
| 1 | (Constant) | ,472 | ,363 |  | 1,299 | ,199 | -,255 | 1,198 |  |  |  |  |  |
|  | MOTrn | ,722 | ,117 | ,654 | 6,186 | ,000 | ,488 | ,955 | ,761 | ,627 | ,511 | ,611 | 1,637 |
|  | MOTfac | ,147 | ,090 | ,173 | 1,635 | ,107 | -,033 | ,327 | ,580 | ,208 | ,135 | ,611 | 1,637 |
| a. Dependent Variable: MOTint | | | | | | | | | | | | | |
